# Supportive and palliative care indicators tool (SPICT™) in a Danish healthcare context: translation, cross-cultural adaptation, and content validation

**DOI:** 10.1186/s12904-022-00931-6

**Published:** 2022-03-24

**Authors:** Heidi Bergenholtz, Anna Weibull, Mette Raunkiær

**Affiliations:** 1 Surgical Department, Holbaek Hospital, Region Zealand, Smedelundsgade 60, 4300 Holbæk, Denmark; 2 REHPA, The Danish Knowledge Centre for Rehabilitation and Palliative Care, Department of Clinical Research, University of Southern Denmark, Vestergade 17, 5800 Nyborg, Denmark; 3 General Medicine, Medical Clinic, Grenaa, Denmark

**Keywords:** Supportive and palliative care indicators tool, Identification, General palliative care, Early palliative care, Translation, Cross-cultural adaptation, Content validation, Palliative care

## Abstract

**Background:**

Early identification of patients who require palliative and supportive care at the general palliative care level is challenging. The Supportive & Palliative Care Indicators Tool (SPICT™) might provide a helpful framework for this process.

**Aim:**

To translate, culturally adapt and content validate SPICT™-DK in hospital, primary care, and general practice and within the broader Danish health care context.

**Methods:**

SPICT™-DK was translated and cross-culturally validated by using the TRAPD-model (Translation, Review, adjudication, pretesting, and documentation) as well as the EORTC- translation guide (European Organisation for Research and Treatment of Cancer). In the pre-(pilot) testing phase, six focus group interviews and five individual interviews were conducted involving *n* = 29 health care professionals from general practice, primary care, and hospital. The qualitative data were analyzed through thematic analysis and the SPICT™-DK was then revised and published.

**Results:**

The interviews revealed that SPICT™-DK can be used to identify people with palliative care needs. Three themes were derived from the analysis and showed SPICT™-DK provides a linguistic framework but must be used as an interdisciplinary tool as that SPICT™-DK requires competencies and collaboration.

**Conclusion:**

SPICT™-DK is now translated and culturally validated in a Danish healthcare setting. The tool is useful to identify people with palliative care needs but must be implemented as an interdisciplinary collaborative intervention. SPICT™ -DK cannot be used by all healthcare professionals as it requires disease-specific competencies. However, it provides a common language for early palliative care interventions which can form the basis for interdisciplinary planning of future treatment and care.

## Background

International studies have shown that patients with life-threatening diseases may benefit from early palliative care to gain improved health-related quality of life and reduced hospitalizations [1–5].

Palliative care is defined by WHO [1] as an approach that aims to improve the quality of life of patients and their families when are facing problems associated with life-threatening illness through early identification and the prevention and relief of suffering through treatment of all symptoms both physical, psychosocial, and spiritual.

However, professionals working in general palliative care i.e. in general practice, primary care (home nursing, home care), and hospitals have challenges in early and timely identification of people with palliative care needs [6–8], and this is especially so when it concerns people with chronic non-malignant diseases [9]. It thus becomes difficult to offer palliative care services to people with life-threatening diseases and their families corresponding to international and national policy recommendations [1, 10, 11]. Internationally, there is no consensus in defining or identifying people who need palliative care [12], and different tools are used to support the identification of patients with palliative care needs [13].

However, in 2017, the Danish National Board of Health issued adjusted recommendations for palliative care, in which the SPICT™ (Supportive & Palliative Care Indicators Tool) was recommended for early identification of patients with palliative care needs [11].

**Fig. 1:**
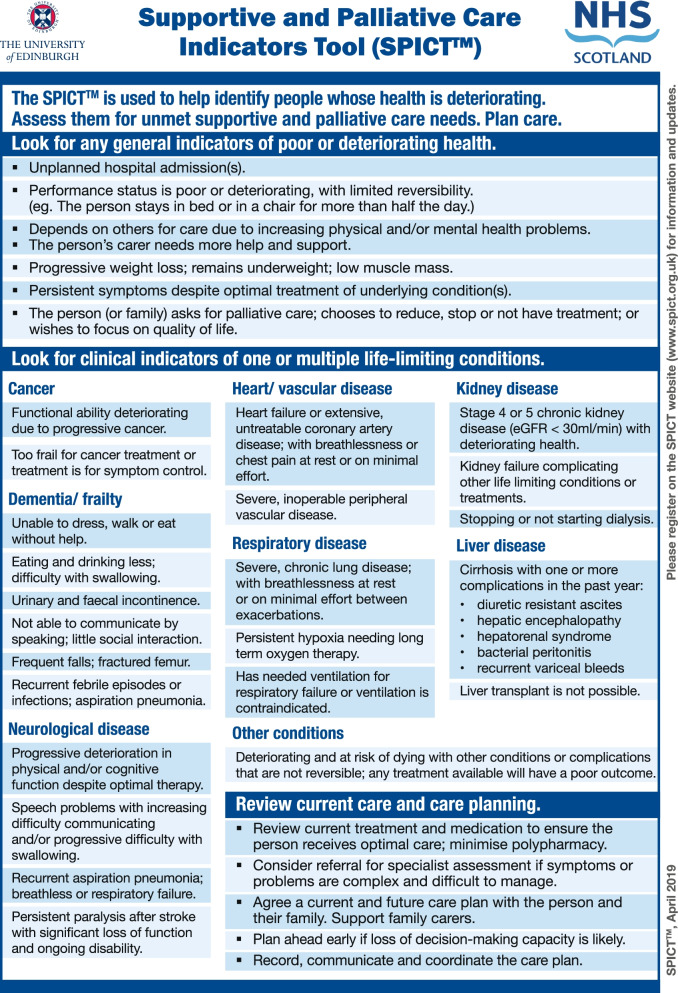
SPICT™

SPICT™ was developed and validated in 2010 by a research team at the University of Edinburgh (Fig. 1). It is a clinical tool supporting health care professionals in the identification of people with deteriorating health conditions and who may benefit from a palliative approach.

SPICT™ was developed to the general palliative level and targets everyone with life-threatening diseases as well as frail older people. Frailty in elderly people may according to Bone et al. [14] be defined as the accumulation of deficits and diminishing reserves, where the frailty state is characterized by an illness trajectory of prolonged dwindling with intermittent episodes of decline. The tool SPICT™ consists of: Six general indicators for deteriorating health and the need for palliative care; seven clinical indicators of one or more life-limiting conditions; and a guide to planning future care and treatment [15]. SPICT™ can be used for all patients, in all contexts, and at any point in the disease trajectory [16, 17]. At present, SPICT™ has been translated into 10 languages [15], for example, German [18], Swedish [19] Italian [20], and Spanish [21], and has been shown to identify people with deteriorating health and those in need of palliative care [22]. For example, studies show that the tool can be useful as a support in clinical decision-making in hospitals to identify patients in need of palliative care [16] and can be used to identify older hospitalized patients at risk of dying within a year [23]. Furthermore, a study by Lunardi et al. 2020 showed [24] that the use of SPICT™ gave a significant improvement in the nurses’ confidence and abilities to recognize patients approaching their end of life.

To our knowledge, little research has been done into the actual implementation and use of SPICT™ and we see in some studies that have an actual call for this task [22], as the tool also has shown to be more time-consuming than for example the surprise question [25].

## Aim/objective

The objective of the study was to translate, cross-culturally adapt and content validate SPICT™ -DK in hospital, primary care, and general practice in the Danish Health Care context.

## Method and setting

This study was initiated by REHPA, The Danish National Knowledge Centre for Rehabilitation and Palliative Care which established a working group consisting of two senior researchers (first and last author) and a general practitioner (2nd author) working in the field of palliative care.

### Translation process

The translation of SPICT™ -DK followed a forward-backward translation and was inspired by the EORTC (European Organisation for Research and Treatment of Cancer) Quality of Life Group Translation Procedure [26] and the TRAPD-model (Translation, Review, adjudication, pretesting, and documentation) [27].

**Fig. 2:**
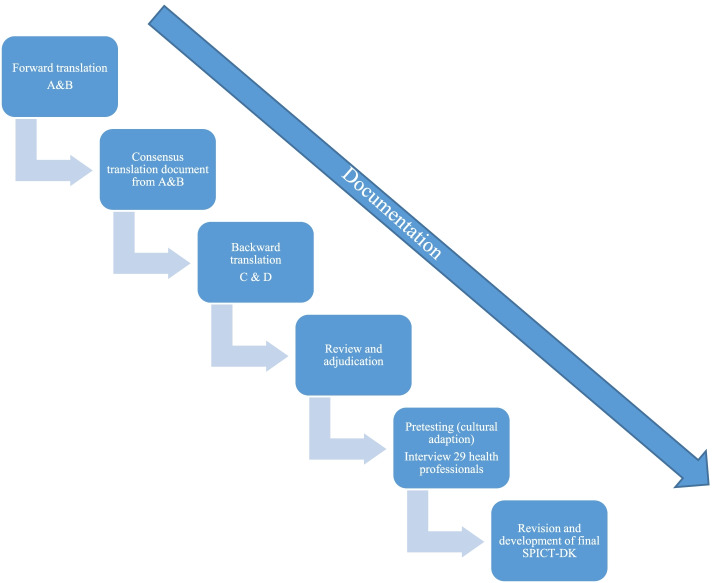
Translation and cultural adaption process

The cross-cultural adaption and content validation [28] was based on interviews with 29 health care professionals from general practice, primary care, and a hospital (Fig. 2).

The translation included native Danish and English translators with special language skills. Table 1 presents the involved actors in the process:

**Table 1:** Involved actors in the translation process

❖ **Translation Coordinator**: First author ❖ **Working group:** All authors, two senior researchers, and a general practitioner ❖ **Forward translators:** From English to Danish (two translators with Danish as mother tongue.) Translator A + B ❖ **Backward translators:** Translates back from Danish to English (two translators with English as mother tongue.) Translator C + D ❖ **Respondents:** A total of 29 health care professionals from the secondary and primary health care sector participated in focus group interviews ❖ **Proofreader**: A professional Danish linguist who performed proofreading on the final Danish translation.	

#### Forward translation

In March 2018 two forward translations of the original SPICT™ were performed by two native Danish-speaking translators with language skills in English as well. They were non-clinicians but had a master’s degree in biology and were therefore familiar with medical terms. The translations were at first performed individually and then a meeting between the translators and the translation coordinator was established and a consensus document was achieved between the translators. The translation coordinator acted as an observer at the meeting and had a special focus on how consensus was reached. Translations from translators A and B were scored ranging from no changes, slightly changes to new translations created in consensus as recommended by Kulis et al. [26].

#### Backward translation

The consensus-translation-document, prepared in the previous phase, was sent to two professional English-language translators, with knowledge of research in the field of palliative care who translated the document back into English.

The translations were then reviewed by the working group, which checked the translations both concerning the Danish translation and the original SPICT™ tool.

#### Review and adjudication

Subsequently, all the translations (from translator A, B, C, and D) were reviewed by the working group, which discussed (un)similarities of both the forward- and backward-translation. Words that did not match the original SPICT™ document were discussed and subsequently resent to the English-language translators (C, D). The SPICT™ -DK tool was adjusted in the working group, and the document thus formed the basis for the interviews with the clinicians.

#### Pretesting (cross-cultural adaption and content validity)

The purpose of the interviews was to test and adjust the translation so that the tool was cross-culturally adapted and had validated content for clinical conditions in Denmark. As Beaton et al. 2000 [29] describe cross-cultural adaptation is necessary to maintain the content validity of an instrument as there exist differences in cultures that a solely linguistic translation cannot conceptualize.

A total of six focus group interviews and five individual interviews (participants = 29) were conducted in spring-summer 2018. For participation in the cross-cultural adaption process, the working group identified one hospital in the Zealand Region, two municipalities in the Capital Region representing two nursing homes, one home care, and one home nursing, and general practices from the Central Jutland Region. The individual interviews were conducted where it was not possible to gather the doctors from the hospital for one focus group interview. Respondents participated voluntarily and were invited: 1. Either by mail and 2; for primary care carers through their immediate supervisor and 3; and through a national website for general practitioners.

The inclusion criteria for the recruited participants were that they were health care professionals and had experience in working with patients with life-threatening diseases.

Table 2 provides an overview of the respondents. In all 12 hospital professionals (five doctors and seven nurses), 12 home care professionals, and five GPs participated. Their work experience went from newly educated to 40 years of experience. Before the interviews, all respondents had read and completed a form, in which each sentence was scored based on the following scale [26]: 1. Hard to answer 2. Confusing 3. Hard to understand 4. Disturbing/offensive.

**Table 2:** Respondents

Respondent	Title	Gender	Experience/year	Place of work	Interview
1	Registered nurse	F	7	Home nursing	Focus 1
2	Registered nurse	F	14	Home nursing	Focus 1
3	Registered nurse	F	24	Home nursing	Focus 1
4	Registered nurse	F	20	Home nursing	Focus 1
5	Health care worker	F	NA*	Home care	Focus 2
6	Health care worker	F	NA*	Home care	Focus 2
7	Health care worker	F	NA*	Home care	Focus 2
8	Registered nurse	F	7	Nursing home	Focus 3
9	Registered nurse	F	9	Nursing home	Focus 3
10	Registered nurse	F	13	Nursing home	Focus 3
11	Health care worker	F	10	Nursing home	Focus 3
12	Health care worker	F	11	Nursing home	Focus 3
13	General practitioner	F	2	General Practice	Focus 4
14	General practitioner	F	7	General Practice	Focus 4
15	General practitioner	M	2	General Practice	Focus 5
16	General practitioner	M	7	General Practice	Focus 5
17	General practitioner	F	15	General Practice	Focus 5
18	Registered nurse/Dialysis	F	15	Hospital	Focus 6
19	Registered nurse/Surgical	F	3½	Hospital	Focus 6
20	Registered nurse/Surgical	F	4	Hospital	Focus 6
21	Registered nurse/Cardiology	F	0,25	Hospital	Focus 6
22	Registered nurse/Geriatrics	F	3	Hospital	Focus 6
23	Registered nurse/Pulmonary	F	22	Hospital	Focus 6
24	Registered nurse/Pulmonary	F	1½	Hospital	Focus 6
25	Doctor/Cardiology	F	20	Hospital	Individual
26	Doctor/Geriatrics	F	20	Hospital	Individual
27	Doctor/Gynocology	F	10	Hospital	Individual
28	Doctor/Nephrology	M	19	Hospital	Individual
29	Doctor/Pulmonary	F	40	Hospital	Individual

^*^NA Not Applicable

The interview began with a review of SPICT™ -DK and the respondents had the opportunity to elaborate on their scores on the form. Subsequently, the focus was on applicability and the open-ended interview questions such as*: “How do you see SPICT™ -DK being used and implemented in your clinical practice?“*A thematic analysis following Braun and Clarke’s six phases [30] was performed. This included an initial understanding of all the interviews; generating initial codes; searching for themes and selecting appropriate extracts. The findings were discussed in the working group and the results from this phase are presented in the results section. This process was used to assess the cross-cultural adaptation [28] and content validity.

After the interviews, the working group revised and developed the final SPICT™ -DK.

#### Documentation

We seek here to present and document the translation and cultural adaption process as transparently as possible, in order to guide future SPICT™ – translations. The SPICT™ -DK was communicated in an internal document in REHPA [31] but has not been published in a scientific context before. The SPICT™ -DK is available from the SPICT™ organization website [32].

### Ethical considerations

The study was registered by the Danish Data Protection Agency (REG-163-2017) and the Declaration of Helsinki [33] was followed. The respondents were informed both verbally and in writing about the project and their participation. They were guaranteed anonymity and confidentiality and they gave informed consent prior to the interview. For this reason, the participants have been anonymized when excerpts are presented in the result section.

## Results

As described in the methods section SPICT™ was translated through a forward-backward translation.

In this result section, we will present the findings from the qualitative thematic analysis which aimed to cross-cultural and content validate SPICT™ -DK.

Three themes emerged from the analysis regarding the SPICT™ -DK content: *A linguistic framework and collaboration tool; suitability for health care professionals; and applicability in health care settings.*

### Theme 1 - a linguistic framework and collaboration tool

This theme contains the perception from the health care professionals who viewed SPICT™ -DK as a relevant tool for their practice and as a linguistic framework for the clinical work they already do when seeing patients with life-threatening chronic diseases. This means that SPICT™ -DK gave a common language for palliative care for the health care professionals. Both the general and clinical indicators from SPICT™ -DK were perceived as relevant and useable.

The health care professionals perceived the content in the SPICT™ -DK – tool, as the content they were dealing with every day but which they had not thought of as palliative care. Therefore, SPICT™ -DK provided a vocabulary for familiar areas in clinical practice:

*«We do a lot of that in advance. But we just do not have words for it »(Respondent 5).*

*«Also in relation to the doctor and especially when it is not our usual doctor. It's something with having a common language »(Respondent 10).*

*«You think it, but you do not put it into words» (Respondent 15)*

These quotes illustrate that the respondents were familiar with the content (general and clinical indicators) of SPICT™ -DK but lacked a vocabulary or language for palliative care. This was independent of the professional group, meaning that both the health care workers, as well as the doctors, shared this notion. We found that several of the respondents described SPICT™ -DK, as a framework for a common language, meaning that SPICT™ -DK could function as a collaboration tool between professionals:

*«The tool (SPICT) could be good for our meetings with the nurses. Because it is not always that the nurses catch our word and vice versa - a collaboration tool to stay focused ». (Respondent 7)*

This was further described as an interdisciplinary tool:

*«Once I have filled it out (SPICT), I will go to the doctor and say: now I have filled this out." (Respondent 19).*

The respondents described how SPICT™ -DK could be a valuable tool in different professional contexts such as staff meetings, supervision, educational contexts, and daily interdisciplinary discussions concerning the initiation of palliative care for patients/citizens.

*«We could use it in a Wednesday teaching. What is it you should look for?» (Respondent 14.)*

### Theme 2 - suitability for health care professionals

We found that was a difference in the suitability between the different professional groups when having to use SPICT™-DK independently. There was a general agreement among the professionals that SPICT™-DK could be used independently by doctors and nurses, however, the doctors perceived the nurses fully capable of understanding the terms:

*«It's not too difficult for the nurses - they will definitely be able to use it. We have a close collaboration with the nurses». (Respondent 15)*

But some of the nurses from primary care formulated challenges in understanding some of the clinical content in SPICT™-DK:

*«We need to look up some of the words*». *(Respondent 9)*

Several respondents expressed that the health care workers could not use SPICT™ -DK independently, because the tool requires an educational level for which these professional groups are not trained:

*«I doubt it (the use of) in nursing homes because they are often health care helpers. I do not think they understand it or they will misinterpret it. It is academically too high a level» (Respondent 14)*

*«It's too complicated for the helpers*» (*Respondent 6)*

However, nurses from the primary sector emphasized that health care assistants and helpers might be able to use parts of SPICT™ -DK that was appropriate to their level of competence:

«*The helpers (in health care) do not know all the words [...] but they can use it where it embraces their level of competence because they do not need to know anything about liver diseases»* (*Respondent 8)*

At the hospital, the professional groups wanted the nursing group to be responsible for the use of SPICT™-DK, but this was challenged due to staff shortages:

*«It is a dream that is far away from us to get any nurses employed and thus able to use the tool» (Respondent 28)*

This led to the third theme on the applicability and implementation of SPICT™-DK.

### Theme 3 - applicability in health care settings

This theme includes respondents’ thoughts on how SPICT™ -DK was applicable in their different health care contexts.

The majority of the respondents considered that SPICT™ -DK was a helpful tool that could raise the quality of palliative care by identifying palliative care needs for patients with life-threatening diseases as well as contribute to planning future treatment and care:

*«Yes, it can be used especially for the latter: “Assess and plan for future care»(Respondent 9)*

Furthermore, it was emphasized that SPICT™ -DK could be useful as an indicator of when health care professionals should initiate «difficult conversations» or more generally take the initiative to talk to the patients and relatives about the course of the disease and the content of such a conversation:

*«It indicates when to talk to relatives and when to talk to the residents: What are the deteriorations? Help assess what the individual needs*» *(Respondent 10)*.

The doctors were more skeptical about using SPICT™ -DK in their practice. On the one hand, some GPs pointed out that SPICT™ indicators could support them in identifying people with palliative care needs:

*«It (SPICT) can be used for outpatient home visits» (Respondent 13).*

*«Just the fact that I have been sitting and reading this, I think I have read some epicrisis (medical journals) where some can be identified» (Respondent 14).*

On the other hand, there was a concern that SPICT™ -DK was merely a tool for creating awareness of the general and clinical indicators and not a scoring tool, where a certain number of scores triggered an algorithm with a description of clinical actions:

*«It is not a scoring tool. I will not use it in my daily life ”(Respondent 15).*

*«I have a hard time figuring it out (the tool)? What then, when I have filled it out does something count more than anything else? What are we going to do after filling in the tool - should I write it in the journal? »(Respondent 20)*

Furthermore, the hospital doctors problematized SPICT™ -DK’s applicability due to time pressure:

*«Our clinical everyday lives right now are so hectic that it's not realistic. It's too much hassle, it's hard to implement » (Respondent 28)*

But also concerns that identifying patients as being characterized as «palliative» could lead to impaired treatment:

*«Regarding the level of treatment - if there is ordered no resuscitation, then you will not even be offered cake for the coffee - you are almost dying».(Respondent 26)*

As described above, most of the respondents were positive about using SPICT™ -DK in practice both in terms of identifying patients and citizens with palliative care needs and planning their future treatment and care. However, the doctors from the hospital especially were more skeptical about the implementation of the tool.

In summary, the results from the interviews which aimed to cross-culturally and content validate SPICT™ -DK shows that the tool is suitable and applicable in a Danish health care context, bearing in mind that the tool should function as interdisciplinary collaboration and a pedagogical device for identifying people with palliative care needs.

#### Revision and development of the final SPICT™ -DK

In the process of translating and cross-culturally validating the SPICT™-DK several discussions about the wording occurred both during the translation process and in the interviews. We will present some of the disagreements on wording below.

For example, the English word “care” was not sufficient when translated to Danish as it was necessary to include that it was both nursing and treatment.

Furthermore, the wording “has needed ventilation for respiratory failure or ventilation is contraindicated” was discussed with an expert doctor in pulmonary diseases and we needed to add a bracket highlighting that it was Non-invasive-ventilation (NIV) and ventilation through a respirator.

Furthermore, the word “carer” was difficult in Danish as it has a double meaning and can apply to both relatives and professional health care workers. Both were included in the Danish version.

Several of the respondents noted that they did not appreciate that SPICT™ was formulated as directives. The working group did not change this form as it was not a challenge that hampered understanding, but merely a preference. The working group assessed that if it were to be changed, it would be too far from the original document. The full report of the translation and all words which were discussed can be found at REHPA’s homepage: https://www.rehpa.dk/wp-content/uploads/2018/12/SPICT-notat-final.pdf [31].

After the interviews, the working group made the final revision of SPICT™-DK and the proofreader performed proofreading on the final Danish translation. The final SPICT™-DK can be downloaded free of charge from the SPICT™ website: https://www.spict.org.uk/the-spict/spict-dk/ [32].

## Discussion

A translation and cross-cultural adaptation and content validation of the SPICT™-DK in general practice, home care, home nursing, and the hospital were completed. Our results show that some clinical and professional words and terms had to be discussed and adjusted to a Danish health care context. This indicates that cultural and linguistic differences do exist among different countries about communication in general and in different health care settings. Thus discussions and considerations in every step of the study were essential for translating and validating SPICT™ into a Danish health care context.

The interviews with the health care professionals show that SPICT™-DK has the potential to identify people with life-threatening diseases in need of supportive and/or palliative care in general practice, nursing homes, and home nursing in Denmark. Furthermore, themes 1, 2, and 3 indicate that SPICT™-DK has the potential to be used as a linguistic framework for supportive and/or palliative care and a collaborative and pedagogical tool among different professional groups and health care settings. In line with a Swedish study [19], our findings indicate that SPICT™-DK has potential in terms of when to take a breakpoint conversation with the patient and the family about future treatment and care.

However, the interview study also revealed certain challenges concerning SPICT™-DK.

Firstly, the hospital staff and general practitioners demanded a scoring schedule where a given number of crosses can predict the need for palliative care. This can be difficult to fulfill, primarily because the definition of palliative care in Denmark and other European countries is based on that of the WHO [34], where it is pointed out that palliative care is a «holistic approach» with a focus on the whole family’s physical, mental, social and spiritual needs and issues. A SPICT™ scoring scheme would not be able to capture this. On the other hand, research studies [35, 36] indicate that a cut-off of two general indicators and one clinical disease-specific indicator predict the need for palliative care and one-year mortality. This may initially be a care mark for clinical practice but it has to be verified in future studies.

Secondly, it was shown that some health care workers probably do not have sufficient professional skills to use SPICT™ -DK in its entirety and alone. Even some nurses had to look up some of the medical terms and phrases in connection with the clinical disease-specific indicators and some doctors expressed that the tool was too complicated. This suggests that although SPICT™-DK looks simple, concise, and logically structured, it requires competencies at a certain level and disease-specific competencies to be able to apply it fully in daily clinical practice. This also specifies that further implementation of SPICT™ -DK, requires introduction and instruction in interpreting, understanding, and using the tool for all involved professional groups. The introduction and teaching must presumably be carried out differentiated depending on the individual professional groups’ competencies and work field and overall in relation to how to use SPICT™-DK as a common collaborative tool. We would like to add that the SPICT-organization has developed a tool called SPICT™-4All for care staff, individuals, and their families/close friends that use non-medical words but are similar to the SPICT™ for health professionals [37]. This might serve as a valuable tool and further research should address the validity of this tool and combination of SPICT™ and SPICT™-4All in a health care setting.

Thirdly, it was noted that the SPICT™ -DK does not guide the entire process of decision-making and assessment of the actual palliative care needs. This implies before using SPICT™ –DK in daily clinical practice it is necessary to clarify that the tool only identifies people who may need supportive and/or palliative care. In addition, it is relevant to consider, whether SPICT™-DK can be used in conjunction with other tools to support future palliative care. To support decision-making ACP (Advanced Care Planning) [38, 39] can be relevant. To support assessment of symptom control can be mentioned IPOS (Integrated Palliative Care Outcome Scale) [40], developed for use among people with advanced diseases and translated to 13 languages (not Danish) or EORTC-QLQ-C15-PAL (translated to Danish), which only is developed for cancer patients with palliative care needs [41]. ACP and EORTC-QLQ-C15-PAL are recommended by the National Danish Board of Health [11]. However, in Denmark, the Danish Health Data Authority has initiated work in 2020 to develop a PRO-palliative care tool to be used at the general palliative level and for people with life-threatening lung-, heart-, kidney-, and cancer diseases [42]. This initially does not include all the target groups covered by SPICT™-DK and especially not people with dementia as well as old and frail people. However, other international PRO-tools can be used to measure the palliative care needs of these target groups - e.g. ESAS (Edmonton Symptom Assessment System), which has been translated into Danish [43].

From this study, we would like to highlight that the key implications for future policy, practice, and research is that SPICT™ –DK can be an important tool for identification and facilitating the planning of future treatment and care. And we see it as a tool that can contribute to the overall optimization of general palliative care in Denmark which should be acknowledged in the policymaking of end-of-life guidelines for clinical practice. Future research must address the implementation of SPICT™ –DK taking into account that it should be used as an interdisciplinary collaborative tool and linguistic framework, but requires training and role- and competence clarifications between the health care professionals.

### Strengths and limitations

The translation, cross-cultural adaptation, and content validation of SPICT™ -DK were successfully performed in this study and we see it as a major strength that this process took place in various health care settings and included health care professionals from general practice, primary care, and hospital. This reflects the settings in which general palliative care is performed. We also see the multidisciplinary sample as a strength, since nurses, health care workers, and doctors should be able to identify patients in need of palliative care, and the knowledge on how these different health care professionals view SPICT™ –DK is valuable for future implementation strategies.

A limitation in our study was that the tool was not directly tested on patients but was a study of the translation, cross-cultural and content validation process. It, therefore, remains to be studied both how the tool is implemented in health care practice as well as how many people with palliative care needs will be identified compared with present work in general palliative care. Furthermore, the gender imbalance of 26 female participants compared to three males is a limitation and may have affected the results in the interviews. However, we see it as a valid representation of how gender is distributed in the health care system in Denmark.

Another limitation is that the respondents expressed that they preferred that the wording in SPICT™ be changed. As the working group chose to see this as personal preference and not something which hampered the understanding, we did not change this as it would change too much from the original SPICT™. However, we acknowledge that this choice may affect future implementation if the tool is viewed as too directive by the health care professionals. However, the other findings from the interviews make us believe that the value gained from integrating SPICT™ -DK as an identification tool will offset this challenge.

## Conclusion

SPICT™ -DK is now translated, cross-culturally adapted, and content validated in a Danish health care context.

The results from this study indicate its use and implementation should address how SPICT™ -DK should be used as an interdisciplinary collaborative and pedagogical tool for identifying people with palliative care needs, in a context where some health care professionals may not have the competence to use it independently. SPICT™ -DK can function as a linguistic framework for the identification of patients with early palliative care needs, but requires a manual on how to use the tool.

Furthermore, it can form the basis for interdisciplinary planning of future treatment and care. We, therefore, see SPICT™ -DK as a valuable tool to promote the overall optimization of general palliative care in Denmark, providing a common frame of reference for when to identify people in need of early palliative care.

## Data Availability

The data material used in this study is available from the corresponding author. The full report on the process can be found at REHPA’s homepage: https://www.rehpa.dk/wp-content/uploads/2018/12/SPICT-notat-final.pdf
